# Parallel vs. comparative evaluation of alternative options by colonies and individuals of the ant *Temnothorax rugatulus*

**DOI:** 10.1038/s41598-018-30656-7

**Published:** 2018-08-24

**Authors:** Takao Sasaki, Stephen C. Pratt, Alex Kacelnik

**Affiliations:** 1 School of Life Sciences, Arizona State University, Tempe, AZ 85287 USA; 2 Center for Social Dynamics and Complexity, Arizona State University, Tempe, AZ 85287 USA; 3 Department of Zoology, University of Oxford, South Park Road, OX1 3PS Oxford, UK; 4 Present Address: Odum School of Ecology, University of Georgia, Athens, GA 30602-2202 USA

## Abstract

Both a single ant and the colony to which it belongs can make decisions, but the underlying mechanisms may differ. Colonies are known to be less susceptible than lone ants to “choice overload”, whereby decision quality deteriorates with increasing number of options. We probed the basis of this difference, using the model system of nest-site selection by the ant *Temnothorax rugatulus*. We tested the applicability of two competing models originally developed to explain information-processing mechanisms in vertebrates. The Tug of War model states that concurrent alternatives are directly compared, so that choosing between two alternatives takes longer than accepting a single one. In contrast, the Sequential Choice Model assumes that options are examined in parallel, and action takes place once any option reaches a decision criterion, so that adding more options shortens time to act. We found that single ants matched the Tug of War model while colonies fitted the Sequential Choice model. Our study shows that algorithmic models for decision-making can serve to investigate vastly different domains, from vertebrate individuals to both individuals and colonies of social insects.

## Introduction

The social insects offer some of the clearest examples of collective cognition, where group members share the processing of information about their environment^1–3^. When presented with two sucrose feeders of different concentration or two nest cavities of different dimensions, insect colonies show consistent preferences that are not simple summations of comparisons made by individual workers^2–4^. Instead, comparison typically emerges from interactions among workers, each having direct knowledge of only one option, and responding to that option according to its quality, so that richer food and better nest sites evoke a higher probability of recruiting nestmates^5–8^ or an increased effectiveness of recruitment^9,10^. Recruited workers follow the same attribute-dependent rules, creating positive feedback that drives colony attention towards the better quality option, ultimately reaching a decision in its favour^2,11–14^.

Collective cognition allows a colony to distribute the burden of option assessment across many individuals, thus minimizing “choice overload.” This phenomenon, first identified in humans, is the worsening of decision quality with increasing numbers of options due to a decision-maker’s limited information-processing capacity^15,16^. For example, isolated workers of the crevice-dwelling ant *Temnothorax rugatulus* show a sharp deterioration in their ability to choose the better of two types of nest cavity when the option set is increased from one good and one poor to four good and four poor cavities. Whole colonies, in contrast, perform at equally high levels regardless of the number of cavities available^17^. The colony’s advantage stems from a reduced assessment load for ants in the social context: each ant in a colony assesses only a small subset of options, whereas isolated ants each visit a larger number of options before deciding^17,18^.

The study of choice overload in *T. rugatulus*, like much research on collective decision-making by animals, has focussed on subjects’ ability to choose the best option, overlooking how much time it takes to do so. However, speed of decision-making is an important measure of performance, and decision makers typically face a trade-off between speed and accuracy, such that they can improve accuracy by spending more time gathering information^19,20^. Some theoretical treatments further imply that larger option sets (i.e. having more options) slow down decision making as well as decreasing the probability of choosing the best option^15,21^.

**Figure 1:**
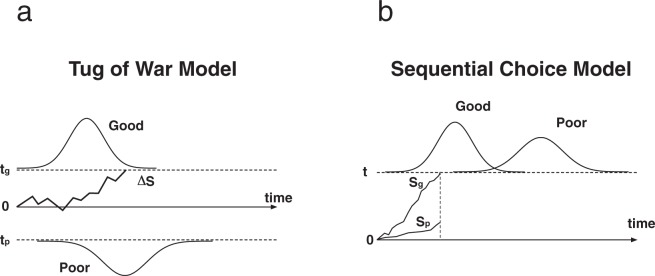
Illustrations of the accumulation of evidence over time for competing options in the tug-of-war model (**a**) and the sequential choice model (**b**). In the ToW model, a decision is made when the difference of signals (ΔS) between the options reaches one of the thresholds (t_g_ or t_p_). In the SCM, each signal accumulates independently, and a decision is made when one of them reaches the single threshold (t). The good and poor curves represent frequency distributions of decision-making latencies observed for each option when it is the only one present. This figure is recreated based on Kacelnik *et al*.^28^.

Examining the temporal component of decision making can also shed light on underlying cognitive mechanisms^22–24^. For example, research on vertebrates has developed information-processing models to understand the relationship between decision speed and accuracy^21,25–27^. Although there is a diversity of such models, they all share common elements. The brain receives streams of noisy sensory evidence for two competing alternatives. It then processes this competing information over time, and, whenever accumulated information reaches a threshold, makes a decision. Two classes of models vary in how decisions depend on the nature of the threshold(s), and consequently in observable predictions: the threshold may require an accumulated difference between competing streams, or each stream may act in parallel, the faster one triggering a decision once it reaches the threshold. The former class of models is known as the Tug of War (ToW), because of the proposed direct interaction between options; it may be most appropriate for animals that must often choose the best among a set of simultaneously present options. The latter model is known as the Sequential Choice Model (SCM), because it assumes a biological decision mechanism adapted to situations where animals mainly meet isolated opportunities and decide whether to pursue them or not^28^. If an animal encounters multiple options simultaneously, the SCM proposes that the decision is reached through a horse race, in which the “choice” results from which option reaches the action threshold earlier, with better (or at least preferred) options reaching the threshold earlier, on average (for details, see Fig. 1).

We test the applicability of these models to decision making in the ant *T. rugatulus*, comparing single ants and whole colonies. One may wonder if these models, which assume that information-processing occurs within the brain of individual animals, are useful to understand colonies, where the decision mechanism is distributed among multiple individuals^28^. We argue that ant colony members, like neurons in an individual brain, collectively process information and reach consensus decisions through their interactions^18,29^. Theoretical models inspired by research in primate brains have already been successfully applied to understand information processing by social insect colonies^30,31^, suggesting that these two conceptual processes, derived from avian research, can also be usefully translated into eusocial insect scenarios.

We evaluate the models by examining decision-making latencies of both colonies and individuals choosing a new home site after their old one has been destroyed. This approach resembles that used in previous work on individual vertebrate decision makers, where subjects are presented one or more targets (typically food items) and the latency until they take one of them is measured. These latencies are typically quite brief (seconds to minutes) and they are used to draw inferences about complex and largely unobservable processes occurring within the animal’s nervous system during this time. For the ants, as will be seen in the results, latencies are much longer (tens to hundreds of minutes), and subjects’ observable behaviour may involve repeated assessment visits to the target nest sites, as well as journeys back to the old nest and, in the case of whole colonies, recruitment communication among workers. In this study, we do not attempt to parse this behaviour into its components, but instead treat the entire series of actions as a unitary decision mechanism. This is analogous to ignoring the neural processes that give rise to the latencies of individual vertebrates, and instead treating the entire latency between target presentation and subject reaction as a single decision event.

The SCM and ToW models differ in their predictions regarding decision latencies in certain situations. Imagine a binary choice, where one option is preferable to the other. According to the ToW model, comparison imposes a time cost, such that latency to choose is longer than if only one option were available. This should be true whether the subject ends by picking the better or worse of the two options. This cognitive evaluation time is consistent with the idea of “choice overload”. In the SCM, in contrast, options ‘compete’ to be chosen by generating a candidate latency independently sampled from the distribution belonging to each option when alone. The option reaching the threshold first becomes the “choice” (see^32^ for a discussion of calling the outcome of such process a choice), and the latency elicited by the not-chosen alternative is simply not expressed or observed. This interaction cross-censors the right tails of both latency distributions, and thus when an option is encountered and chosen in the presence of an alternative, observed mean latencies are shorter than those for the option when presented alone^28^. The fact that the less preferred option experiences more severe cross-censoring (only the extreme of its left tail sees action) leads to one additional prediction in the SCM: the predicted shortening of latency for binary respect to single-option choices is more pronounced for the worse (less preferred) option than for the better (preferred) option. As noted above, the ToW model predicts a shift in the opposite direction (i.e. longer latencies to action when there are two options rather than one), but it makes no specific prediction for whether the added time should differ between options. Table 1 summarises these predictions.

**Table 1:** Comparison of experimental results with predictions of the Tug-of-War (ToW) model and the Sequential Choice model (SCM).

	ToW	SCM	Results for individuals	Results for colonies
**Pooling Poor and Good sites**	Longer in binary, due to comparative evaluation	Shorter in binary, due to cross censorship	**Longer in binary (ToW)**	**Shorter in binary (SCM)**
**Only Poor site**		Much shorter in binary, due to strong cross censorship	No difference	**Shorter in binary (SCM)**
**Only Good site**		Weakly shorter in binary, due to weak cross censorship	**Longer in binary (ToW)**	No difference

The second and third columns show each model’s expected differences between the latency to choose an option from a binary set and the latency to take the same option when it is the only one available. The fourth and fifth columns show the latency differences actually observed for individuals and colonies, respectively. Note that latencies of decision making are compared only within each subject group (individuals or colonies). Significant latency differences (p < 0.05) are shown in boldface, with the supported model indicated in parentheses.

Which model might we expect to hold in nest site choice by ants, at both individual and colony levels? The collective decision process in *T. rugatulus* colonies is largely driven by the recruitment behaviour of worker ants, each with knowledge of only one candidate nest^17,18^. This distributed assessment process suggests that the parallel and independent option evaluation hypothesised by the SCM can potentially match the decision-making process for colonies. In contrast, when individual ants are experimentally isolated from their colonies, they are deprived of informational input from other scouts, and may be forced to compare qualities of two sites by visiting both^17,33^. This may cause decisions between two alternatives to take longer than acting towards a single one, in concordance with the ToW model.

We separately analysed whole colonies and isolated workers of *T. rugatulus*, to determine which of these two kinds of model best accounts for decision processes at each level. Subjects (either individuals or colonies) were presented with a nest site decision in one of three potential situations: a single good cavity, a single poor cavity, or a binary choice between both nest types. Good and poor nests differed only in diameter of the entrance hole, based on previous studies showing that *Temnothorax* have strong preference for smaller entrances^18,34,35^. In all cases we measured the duration between option presentation and emigration to a new site as a decision latency, and compared the results with the models’ predictions.

## Results

**Figure 2:**
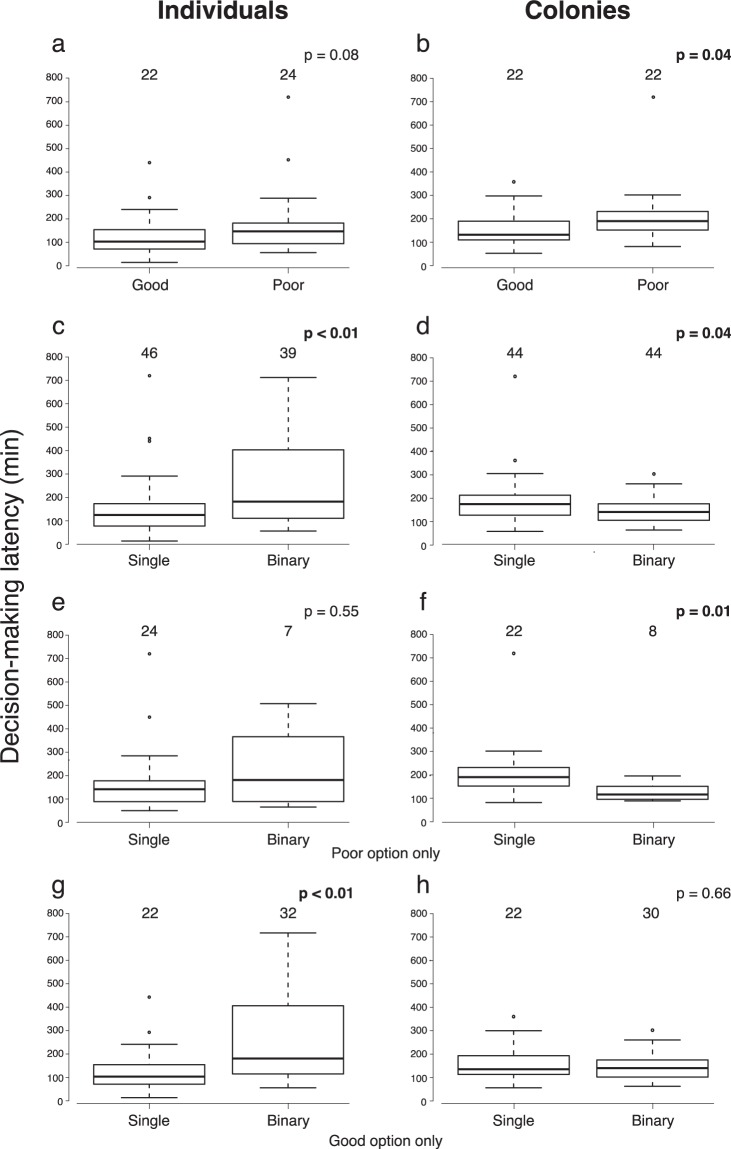
Decision-making latencies for individuals (left column) and colonies (right column). (**a**,**b**) comparison between the good option and the poor option when a single option is present, (**c**,**d**) comparison between the single option condition and the binary choice condition, (**e**,**f**) comparison between the single option condition in which the poor option was present and the binary choice condition in which subjects chose the poor option, (**g**,**h**) comparison between the single option condition in which the good option was present and the binary choice condition in which subjects chose the good option. Each box extends between the lower and upper quartiles, a horizontal line within the box indicates the median, and whiskers show the range of the data, except for outliers indicated by open circles. Each number above a box is a sample size for the group. Within each panel, the latencies were compared using a Mann-Whitney U test (significant p values are shown in bold).

We first tested if the nest with a smaller entrance (good nest) was accepted faster than the one with a larger entrance (poor nest) even when there was no comparison. This was confirmed: when a single nest was present, both individuals and colonies made faster decisions (defined operationally as completing the transport of the brood, see Methods) for the good than for the poor site (for individuals p = 0.07, Fig. 2a; for colonies p < 0.01, Fig. 2b) although the difference for individuals was marginally below conventional statistical significance. This result is consistent with prior studies reporting faster migration to better nest sites^12,18,36^.

When both nest types were present, individuals took significantly longer to make a choice than they did in the presence of a single option of either type (p = 0.04), consistent with the ToW model (Fig. 2c). Colonies, in contrast, took a significantly shorter time for the binary choice than they did when given a single nest (p = 0.04), thus agreeing with the SCM (Fig. 2d).

We then tested the SCM’s prediction that in binary presentations in which the poor nest was chosen latencies should be shorter than when only a poor nest was present. The poor nest was chosen in about 16% and 18% of binary cases for individuals and colonies, respectively. We found no significant (p = 0.54) difference in latency for individuals (Fig. 2e), but for colonies the comparison showed significantly (p = 0.01) faster choices in the binary than in the single option condition (Fig. 2f).

We made the same comparison for cases in which the good option was chosen, to test the SCM’s prediction of a weaker difference between the binary and single-nest cases when the good nest is selected. In this case while individuals made significantly (p < 0.01) faster decisions when only the good nest was present than when facing the binary choice (Fig. 2g), colonies did not show any significant differences between these conditions (p = 0.71; Fig. 2h). The results are summarised in Table 1.

To ensure that the observed results were robust with respect to assumptions regarding latency distributions, we subjected the same data to a survival analysis (Supplementary Information). The observed patterns remained the same.

## Discussion

Our experimental results showed a contrast between colonies and individuals in the performance of the two models under test. While neither model offers a perfect description of the data, results for individual ants are better accounted for by the ToW model, while those for colonies show better agreement with the SCM (Fig. 2). In particular, individuals presented with two options took more time to decide than they did when presented with only one option, while colonies made faster choices in the binary condition. In other words, our empirical results fit the predictions derived from two competing models (Table 1).

Both models are simplifications aimed at highlighting specific issues, and leave aside other processes known to participate in nest site selection. They nonetheless capture interesting and previously overlooked features of eusocial organisation. For example, individual scouts acting within colonies typically evaluate a single option, with the colony integrating multiple parallel assessments by independent scouts^17,18,33,36^. The colony’s choice is thus based on the actions of individual ants that do not have the opportunity to make the type of direct comparison that is responsible for choice overload according to the ToW model.

It is worth noting that having the ability to make parallel comparisons does not necessarily force colony level decisions to follow a horse-race process such as embodied in the SCM. Interactions among nest site scouts could generate evaluation time costs. For example, inhibitory interactions, in which a scout committed to one site diminishes the effectiveness of recruiters to competing sites, could produce the time cost at the heart of choice overload and predicted by the ToW model. In theory, the presence of multiple nest sites could enhance competition between scouts and lead to longer decision times, but this does not occur in our data. There is evidence for this kind of inhibition during nest site selection by honeybees, but it is less clear whether *Temnothorax* possess analogous mechanisms. In laboratory migrations, *T. albipennis* scouts sometimes mark a poor nest in a way that reduces the colony’s likelihood of later choosing it^37^. There is no direct evidence that *Temnothorax rugatulus*, the subject of our experiments, does something similar, but a scout that is disturbed in a potential nest site marks it with a pheromone from its mandibular glands that has the effect of reducing the site’s attractiveness^37^. It is not yet known whether the ants deploy this signal in the normal course of colony migration. The decision latency evidence presented here does not support a role for such inhibitory interactions, but it would be premature to conclude that these ants do not use these pheromones or some other inhibitory signal. It may be that they are deployed only seasonally, as appears to be the case for *T. albipennis*^37^, or only for sites worse than those presented here. In any event, if such signals do play a role, their consequences are not sufficient to override the fast decisions that derive from processing multiple options in parallel.

Similarly, it is not necessarily true that individual decisions must follow the ToW model, and could never fit the predictions of the SCM, because of being unable to make simultaneous comparisons. In fact, the simplest “memoryless” model^38,39^ for individual decision making predicts the opposite. In one such model, an ant makes a series of visits to competing nests, ending each visit with a decision to accept the present nest site and settle or reject it and continue searching. Since the ant retains no memory of each site, her decision-making process can be considered as a series of independent Bernoulli trials (accept or reject). Under this assumption, it can be shown that the ant counterintuitively fits the predictions of the SCM, making fewer visits before settling in potential nest sites in the binary condition than in the single option condition. This is always true for the poorer cavity and also true for the better cavity for a wide range of option sets (see Supplementary Information for a detailed analysis of this model). Our empirical results instead show the opposite pattern, with individual ants making slower decisions in the binary case (Fig. 2c). This suggests that ants do retain memory of visited nests during a migration, as previous studies have also shown^17,33,36^.

Individual ants showed clear evidence of a time cost when comparing two options as opposed to settling into a singly available one. This is consistent with earlier findings that showed a greater ‘choice overload’ effect on individual ants than on colonies. For example, single ants choose appropriately (i.e. mostly pick the better site) when facing two candidate nest sites, but drift towards random when choosing between the same nest types in two sets of four. Colonies instead continue to choose appropriately when facing the same enlarged set^17^. Individuals are also vulnerable to decoy effects in choices among options that vary in multiple attributes^33^, while whole colonies are more resilient towards decoys, consistently adhering to economic principles of rational choice^33,40^. These differences suggest that sharing the burden of information gathering and decision-making across multiple individuals has benefits that are not always immediately obvious: colonies rely on adaptive satisficing without suffering major increases in the probability of errors, while individuals face the full conflict between accuracy of choice and time to decision.

Given the obligatory eusocial condition of our subject species, the functional significance of decisions by isolated workers deserves some caveats. It is rare for a worker to make decisions in isolation of its colony, nor are individual worker ants evolved to maximise fitness as singletons, but their behavioural mechanisms do influence consensus building and are worth investigating. Direct comparison of more than one nest site by a single ant may be particularly relevant when colonies have split between sites and would benefit by reuniting^41^. It has been argued that the highly distributed nature of colonial decision-making has led individual ants to lose their ability to make direct comparisons^38,42^. Our results add to growing evidence that it is not the case and that individuals do retain the capacity to make such comparisons^17,33,36^.

The idea of collective cognition analogizes distributed information processing by a group with the activity of a single brain^43–46^. Our study supports this analogy by showing that the collective decisions of an insect colony can be contrasted with predictions of the kinds of models developed to deal with choice processes by individual vertebrates^13,31,47,48^. For example, and according to current evidence, starlings facing a choice between two foraging alternatives deploy mechanisms evolved for handling individual opportunities sequentially, without a dedicated comparative evaluation process^28,49^. Our results show that an ant colony choosing between two nest cavities also treats the alternatives independently, but the colony has the capability of distributing the information gathering between workers. The consequence, however, is similar to that in starlings: greater option number leads to faster, rather than slower decisions, and neither colonies of the ant *T. rugatulus* nor individual starlings experience choice overload.

## Materials and Methods

### Nest Designs

We use the descriptors ‘good’ and ‘poor’ to refer to sites with small (2 mm diameter) and large (5.5 mm diameter) entrances, respectively. Laboratory tests have shown that, over this range of sizes, *Temnothorax* have a strong and consistent preference for the smaller entrance^17,34,50^. Nest cavities were composed of glass microscope slides (50 × 75 mm) above and below a balsa wood slat. A circular cavity (38 mm diameter) was cut through the middle of the slat, and a round entrance hole was drilled through the centre of the roof^17^. Subjects started each experimental trial in a standard home nest with an intermediate entrance size (3.7 mm).

### Subjects

Ninety colonies of *Temnothorax rugatulus* were used for colony-level tests. An additional 29 colonies provided 92 worker ants (1–6 ants per colony) for the individual tests. We selected active nest site scouts using a previously described procedure^33^. All colonies had at least one queen, with worker populations ranging from 92 to 193 and brood populations ranging from 53 to 280 (detailed information on the subjects can be found in Supplementary Data). Colonies were collected in the Pinal Mountains near Globe, Arizona (N 33° 19.00′, N 110° 52.56′, W). Each colony was housed in a nest like those described above. Nests were kept in a plastic box (11 cm × 11 cm) with Fluon-coated walls. Each box was provided with a water-filled plastic tube capped with cotton and an agar-based diet that was refreshed weekly^51^.

### Procedure

**Figure 3:**
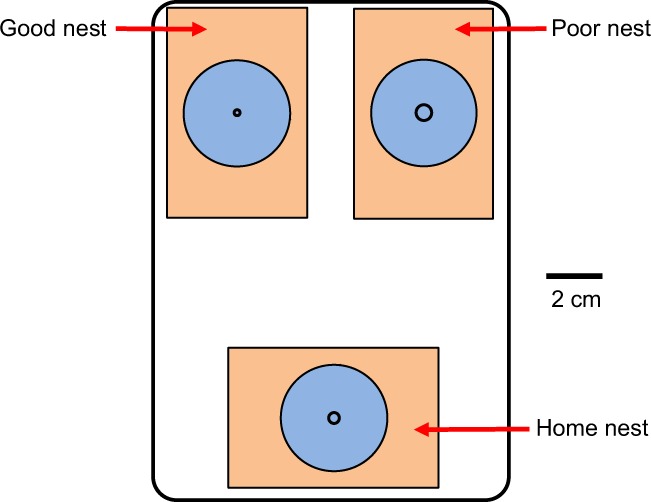
Experimental arena for the binary choice condition. The single option conditions used a similar setup, but only one of the two target nests (either good or poor) was present. In each test, subjects started in the home nest and were induced to move to a new cavity. The good nest has a smaller entrance (2 mm) than the poor nest (5.5 mm). The locations of the target nests were randomized in each test.

We measured decision-making latency in three settings: (1) a single good nest was present, (2) a single poor nest was present, and (3) both nest types were present (Fig. 3). Before each test, the subject (individual or colony) was induced to move into a home nest placed within an experimental arena (an 18 × 13 cm plastic tray whose walls were coated with Fluon). For tests on individuals, we deposited three brood items just outside of the home nest and introduced a single ant to the arena. The ant was judged ready for testing once it had transported the brood into the home nest. For tests on colonies, we placed the colony, still housed in its original nest, adjacent to the home nest. We then removed the roof of the original nest to induce emigration. The original nest was then removed from the arena. Seven ants and two colonies failed to migrate to the home nest and were not tested further.

The subject was then presented with a choice set (either a good nest alone, a poor nest alone, or one nest of each kind), and induced a migration by removing the roof of the home nest. Decision-making latency was defined as the time between removal of the home nest roof and transport of the last brood item to the new site. For binary choices (i.e. when two cavities were presented), we also noted which was chosen. Individual ants always showed an unambiguous choice by transporting all brood into one site. Colonies, however, sometimes split between sites. If one site contained all queens and brood items, as well as more than 90% of colony members we designated that as the colony’s choice. This criterion was not met in 6 out of 44 binary choice trials. These data were not included in the analysis of comparing latencies within each nest type between the single option condition and the binary choice condition (Fig. 2f,h). In another 4 trials (2 individuals and 2 colonies), the subject failed to complete a migration within 12 h. In such cases, their decision-making latencies were considered as 12 h. To check for the bias of these censored data, we also analysed the data using a survival analysis (see below for details).

Before each experiment, all glass slides were washed in a commercial dishwasher, and the experimental arena was cleaned with ethanol. Balsa slats were made fresh for each experiment and never reused. Each subject was tested only once (i.e. experienced only one choice set).

### Analysis

Decision-making latencies were log-transformed and compared using a general linear mixed model. For the colony analyses, we included numbers of workers and brood items as random effects. There were no significant effects of the numbers of workers and brood items for any of the colony analyses (p > 0.05). The normality of residuals and the equality of variances were checked using a Shapiro’s test and a Levene’s test, respectively. The assumptions for a linear model were met for all the tests (p > 0.05 for Shapiro’s and Levene’s tests), except the poor nest comparison in colonies (Fig. 2f) where the normality of residuals was violated. We also tested this comparison using a Mann-Whitney test and confirmed the same statistical result (W = 33, p = 0.01).

For the individual data, some colonies provided multiple ants, creating a potential problem of pseudoreplication if ants of the same colony share idiosyncrasy. To avoid this, we calculated the average latency across ants originating from the same colony within the same treatment and treated the average as a single data point.

Some data were censored (i.e., subjects made no decision within the 12 h duration of the experiment), potentially biasing the results of the Mann-Whitney tests. To check for this, we also analysed the data via survival curves fit using the Cox proportional-hazards regression model, which can account for censored cases (Supplementary Information). All statistical tests were performed in R version 3.0.1. Data and R scripts are available in Supplementary Data and Supplementary Information, respectively.

## Electronic supplementary material

Supplementary Information
