# Supplementary material for: Parallel vs. comparative evaluation of alternative options by colonies and individuals of the ant Temnothorax rugatulus
Source: Sci Rep. 2018 Aug 24;8:12730. doi: 10.1038/s41598-018-30656-7 (PMC6109163; doi:10.1038/s41598-018-30656-7)
Supplement: Supplementary file 1 — Supplementary Information [file 41598_2018_30656_MOESM1_ESM.docx]

**Supplementary Information**

**“Parallel vs. comparative evaluation of alternative options by colonies and individuals of the ant *Temnothorax rugatulus*”**

**Takao Sasaki, Stephen Pratt and Alex Kacelnik**

***Survival analysis of decision latencies***

Decision-making latencies were analysed via survival curves fit using the Cox proportional-hazards regression model. We compared the two single option conditions, the chosen nests (good vs. poor) of the binary condition, and the conditions (single option vs. binary) within each subject group (individual or colony). For the colony data (Figure S1b, d, f and h), we included the number of workers and brood items as fixed covariates. These covariates had no significant effects in any tests (Table S1) and thus were removed in further analysis. If analysis includes the subjects’ choices in the binary condition, the data of undecided and split choices were excluded from the analysis because we were not able to categorize them according to their choices.

*Results*

When a single nest was present, both individuals and colonies made faster decisions when the nest was good (small entrance) than when it was poor (Figure S1a, b). These differences must be interpreted cautiously, as they were marginally below conventional statistical significance (for individuals p = 0.05; for colonies p = 0.07). Still, the observed difference matches prior studies reporting faster migration into better nest designs^1-3^.

When both nest types were present, individuals took significantly (p < 0.01) longer to make a choice than they did in the presence of a single option, consistent with the ToW model (Figure S1c). Colonies, in contrast, took a significantly (p < 0.05) shorter time for the binary choice, thus agreeing with the SCM (Figure S1d). When we considered only those binary choices in which subjects chose the poor option (about 16% and 18% of cases for individuals and colonies, respectively), we found no significant (p = 0.72) difference in time to decision for binary vs. single choices by individuals, a result that does not help in discriminating between the models (Figure S1e). For colonies, on the other hand, the same comparison showed significantly (p < 0.01) faster choices in the binary condition (Figure S1f), which accords with the SCM. Finally, when comparing only those binary choices in which subjects chose the good option (about 84% and 82% of cases for individuals and colonies, respectively), latencies to choose by individuals were shorter (p < 0.01) in the single condition than in the binary condition, which is consistent with the ToW model (Figure S1g). Colonies showed no significant (p = 0.54) difference (Figure S1h), an outcome that does not help to discriminate between the models. Notice, however, that according to the SCM the shortening of latencies between single option and choice trials is expected to be more marked for trials when the poor option is chosen than when the better one is chosen, which is consistent with the difference observed. In sum, similar to the results of the Mann-Whitney tests, these results show that decision making of individual ants matches with the ToW model, while that of colonies agrees with the SCM.

For the analyses above, we marked the timing of each decision as the transport of the last brood item from the original home nest to the chosen site. This criterion treats the process of transporting the colony to the nest site as part of the decision process. In this species, the colony's decision emerges from the tandem runs and transports that move the colony, rather than being a process internal to each ant that is subsequently implemented via recruitment behaviour. As a consequence, the outcome of a choice is not reliably signalled by the first recruitment act or the first brood transport: decision-making is a competitive process in which partial levels of recruitment may occur to multiple sites. Operationally, we considered the decision complete only when recruitment essentially ended. Both adults and brood are transported by workers to new potential nests, but we focussed on brood because adult transport often continues long after the old nest is empty, apparently due to the retrieval of lost workers wandering the arena. To test the robustness of our conclusions, we repeated the analysis of collective decision-making with an alternative criterion: the completion of the fifth brood transport, a milestone that is generally reached very early in the transport phase of migration. The results show the same basic pattern, except that the significance of the shortening of latencies for single poor options vs. binary poor options declined considerably (p = 0.09; Figure S2). Individual decisions were analysed only using transport of the last brood item as a criterion; with only three brood items to move, ants generally completed transport within a short period of time, hence the choice of criterion had little effect on measured latencies.

***A model of “memoryless” ants that accords with the SCM***

Here we address the argument that the SCM cannot conceivably apply to individual ants, because they do not make simultaneous comparisons of multiple options, but instead consider each one in turn. We develop a mathematical model showing that under reasonable assumptions individual ants can make decisions that fit the predictions of the SCM (i.e. the ant takes longer time to accept a nest in the single option condition than in the binary choice condition, the opposite of what our results show). This model shows that the SCM for individual ants is a viable hypothesis worth being tested empirically, as we do in the main body of the paper.

First we consider the case where only a single site is available, either good or poor (Figure S3a). The ant searches the arena, and upon encountering the site she assesses it and decides either to reject it and go on searching or to accept it, with probability *P_g_* or *P_p_*, for good and poor sites, respectively (*P_g_* > *P_p_*). If she rejects the site and then encounters it again, she repeats the same process, having retained no memory of her previous visit. The ant will eventually accept the site, and her probability of doing so after *n* visits is given by:

$f\left( n \right)={P_{i}\left( 1-P_{i} \right)}^{\left( n-1 \right)}$ (*i* = *g* or *p*) (1)

The expected number of visits until she accepts is

$E_{i}^{1}=\frac{1}{P_{i}}$ (2)

where the superscript ‘1’indicates that only one site is available.

We next consider the binary condition (Figure S3b). The probability *P_a_* that the ant accepts *either* nest site in each visit is the arithmetic mean of *P_p_* and *P_g_*:

$P_{a}=\frac{P_{p}+P_{g}}{2}$ (3)

The probability *P_r_* that the ant accepts *neither* nest sites in each visit is:

$P_{r}=1-P_{a}$ (4)

The probability that the ant accepts a nest site after *n* visits is:

$f\left( n \right)=P_{a}{P_{r}}^{\left( n-1 \right)}$ (5)

The process will end with acceptance of either the poor nest or the good nest. We first consider the case that the poor nest is chosen. If we assume that the two sites are equally likely to be encountered, then the probability that the ant accepts the poor site after *n* visits is:

$f_{p}\left( n \right)=\frac{1}{2}P_{p}{P_{r}}^{\left( n-1 \right)}$ (6)

From this we can calculate the expected number of visits until the poor nest is accepted:

$E_{p}^{2}=\sum_{n=1}^{\infty} n\frac{1}{2}{{P_{p}P}_{r}}^{\left( n-1 \right)}=\frac{1}{2}P_{p}\sum_{n=1}^{\infty} n{P_{r}}^{\left( n-1 \right)}$ (7)

This is simply the sum of each possible value of *n* multiplied by its probability of occurrence. To calculate this sum, we note that the sum of an infinite geometric series can be expressed as follows:

$\sum_{n=1}^{\infty} nx^{n-1}=\frac{1}{\left( 1-x \right)^{2}}$ (8)

Thus, the expected number of visits is:

$E_{p}^{2}=\frac{1}{2}P_{p}\sum_{n=1}^{\infty} n{P_{r}}^{\left( n-1 \right)}=\frac{1}{2}P_{p}\frac{1}{\left( 1-P_{r} \right)^{2}}$ (9)

Using equations 3 and 4, we express *P_r_* in terms of *P_p_* and *P_g_*:

$E_{p}^{2}=\frac{1}{2}P_{p}\frac{1}{\left( 1-\left( 1-\frac{\left( P_{p}+P_{g} \right)}{2} \right) \right)^{2}} =\frac{2P_{p}}{\left( P_{p}+P_{g} \right)^{2}}$ (10)

We can see that $E_{p}^{1}>E_{p}^{2}$ (i.e. the number of visits is larger in the single option condition than in the binary choice condition) if:

$\frac{1}{P_{p}}>\frac{{2P}_{p}}{\left( P_{p}+P_{g} \right)^{2}}$ (11)

By rearranging this, we get:

$\frac{1}{2}>\left( \frac{P_{p}}{P_{p}+P_{g}} \right)^{2}$ (12)

Because *P_p_* < *P_g_*, this is true by definition.

The same reasoning can be applied to the good nest site:

$\frac{1}{P_{g}}>\frac{{2P}_{g}}{\left( P_{p}+P_{g} \right)^{2}}$ (13)

This can be rearranged:

$\frac{1}{2}>\left( \frac{P_{g}}{P_{g}+P_{g}} \right)^{2}$ (14)

$\sqrt{\frac{1}{2}}>\frac{P_{g}}{P_{g}+P_{b}}$ (15)

$\sqrt{\frac{1}{2}}P_{g}+\sqrt{\frac{1}{2}}P_{b}>P_{g}$ (16)

$P_{g}\left( 1-\sqrt{\frac{1}{2}} \right)<{\sqrt{\frac{1}{2}}P}_{b}$ (17)

$\frac{P_{g}}{P_{b}}<\frac{\sqrt{\frac{1}{2}}}{1-\sqrt{\frac{1}{2}}}=2.41$ (18)

In other words, adding a poor nest reduces decision time too, as long as the good nest is no more than 2.41 times better than the poor one. Thus, over a wide range of option qualities for two competing nest sites, the memoryless ant model accords with the SCM’s prediction that time to decide should be shortened by the presence of an alternative. The fact that this is not what the data show (see main text) implies that some or all of the model’s assumptions must be rejected.

Figure S1. Decision-making latencies for individuals (left column) and colonies (right column). Latencies are represented as survival curves showing the decline in number of undecided subjects over time (faster decisions give steeper declines). Blue and red lines show decisions in favour of the poor and good sites, respectively. Purple lines show both decision outcomes combined. Solid and broken lines indicate the single option and binary choice conditions, respectively. Within each panel, the survival curves were compared via Cox proportional hazards models (significant p values are shown in bold).

Figure S2. Decision-making latencies for carrying the 5^th^ brood item in colonies.

(a)


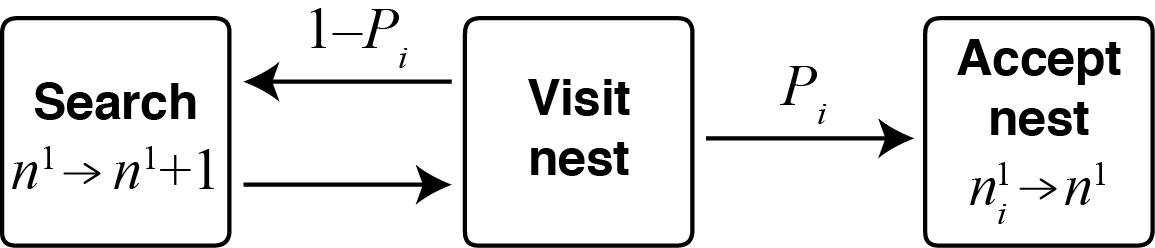


(b)


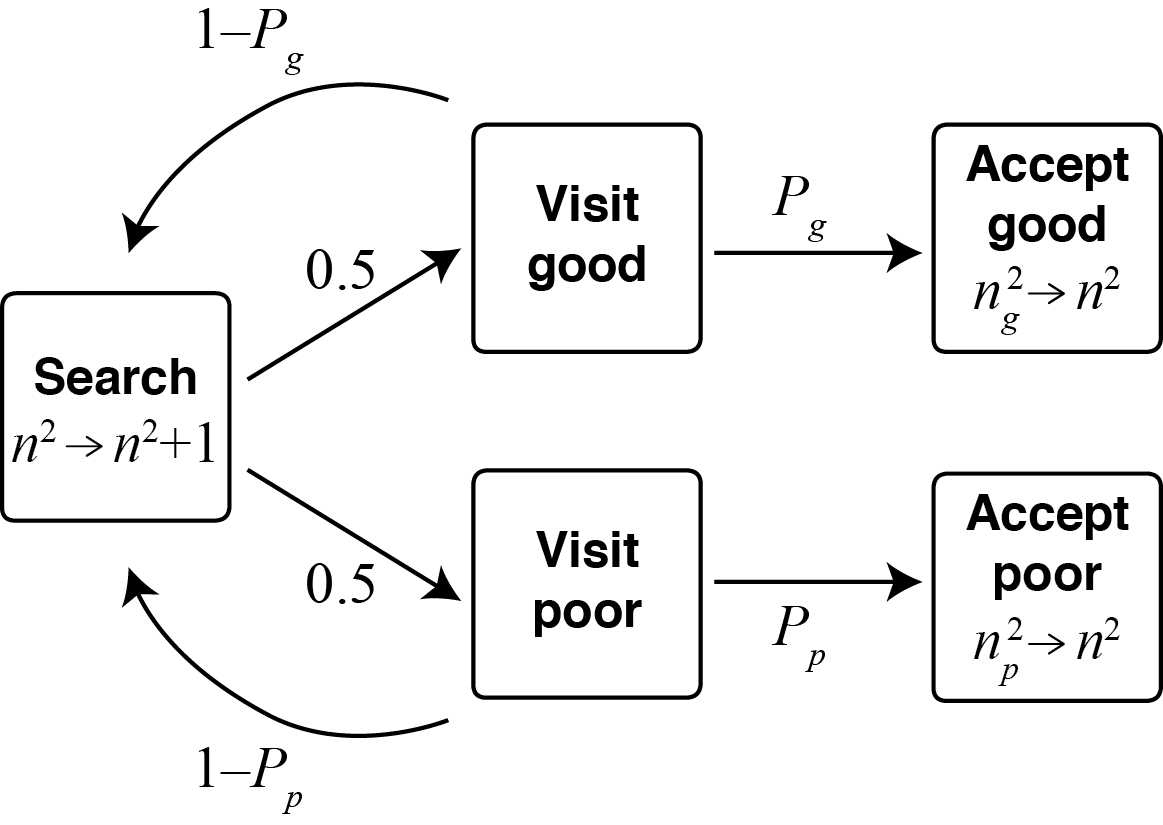


Figure S3. Proposed decision-making process for solitary ants in a) the single option condition and b) the binary choice condition. Each box is a discrete state and each arrow indicates a possible transition, accompanied by a parameter giving the transition probability. Because the transition probabilities depend only on the current state, this process is “memoryless”.

| Table S1. The results of brood and worker effects in the survival analysis. In all the tests, the number of brood items and workers do not have significant effects on decision-making latencies.  Single option: poor vs. good (Figure 1Sb) | | | | | |
| --- | --- | --- | --- | --- | --- |
|  | coefficient | exp(coef) | se(coef) | z | Pr(>\|z\|) |
| nest type | 0.5406263 | 1.7170819 | 0.317527 | 1.703 | 0.0886 |
| worker | -0.0009317 | 0.9990687 | 0.0046718 | -0.199 | 0.8419 |
| brood | 0.0027343 | 1.002738 | 0.0032983 | 0.829 | 0.4071 |
|  |  |  |  |  |  |
| Single option vs binary choice (Figure 1Sd) | | | | | |
|  | coefficient | exp(coef) | se(coef) | z | Pr(>\|z\|) |
| condition | 0.5108171 | 1.6666524 | 0.2255124 | 2.265 | 0.0235 |
| worker | 0.0007277 | 1.0007279 | 0.0030494 | 0.239 | 0.8114 |
| brood | 0.0022625 | 1.002265 | 0.0022269 | 1.016 | 0.3096 |
|  |  |  |  |  |  |
| Single option poor vs binary choice poor (Figure 1Sf) | | | | | |
|  | coefficient | exp(coef) | se(coef) | z | Pr(>\|z\|) |
| condition | 1.287027 | 3.622003 | 0.493302 | 2.609 | 0.00908 |
| worker | 0.006333 | 1.006353 | 0.005302 | 1.195 | 0.23226 |
| brood | -0.002188 | 0.997814 | 0.004243 | -0.516 | 0.60612 |
|  |  |  |  |  |  |
| Single option good vs binary choice good (Figure 1Sh) | | | | | |
|  | coefficient | exp(coef) | se(coef) | z | Pr(>\|z\|) |
| condition | 0.182645 | 1.200388 | 0.306235 | 0.596 | 0.551 |
| worker | -0.001163 | 0.998837 | 0.004096 | -0.284 | 0.776 |
| brood | 0.004007 | 1.004015 | 0.002989 | 1.341 | 0.18 |

**References**

1. Pratt, S. C. & Sumpter, D. J. T. A tunable algorithm for collective decision-making. *Proc Natl Acad Sci USA* **103,** 15906–15910 (2006).

2. Mallon, E. B., Pratt, S. C. & Franks, N. R. Individual and collective decision-making during nest site selection by the ant *Leptothorax albipennis*. *Behav Ecol Sociobiol* **50,** 352–359 (2001).

3. Sasaki, T., Granovskiy, B., Mann, R. P., Sumpter, D. J. T. & Pratt, S. C. Ant colonies outperform individuals when a sensory discrimination task is difficult but not when it is easy. *Proc Natl Acad Sci USA* **110,** 13769–13773 (2013).

4. Kacelnik, A., Vasconcelos, M., Monteiro, T. & Aw, J. Darwin’s “tug-of-war” vs. starlings’ “horse-racing”: how adaptations for sequential encounters drive simultaneous choice. *Behav Ecol Sociobiol* **65,** 547–558 (2011).
